# Comparative cytogenetic analysis of the germline-restricted chromosome in Fringillidae species (Passeriformes, Aves)

**DOI:** 10.18699/vjgb-26-48

**Published:** 2026-05

**Authors:** L.P. Malinovskaya, K.V. Tishakova, P.M. Borodin

**Affiliations:** Novosibirsk State University, Novosibirsk, Russia Institute of Cytology and Genetics of the Siberian Branch of the Russian Academy of Sciences, Novosibirsk, Russia; Novosibirsk State University, Novosibirsk, Russia Institute of Molecular and Cellular Biology of the Siberian Branch of the Russian Academy of Sciences, Novosibirsk, Russia; Institute of Cytology and Genetics of the Siberian Branch of the Russian Academy of Sciences, Novosibirsk, Russia

**Keywords:** germline-restricted chromosome, GRC, chromosome evolution, fluorescent in situ hybridization, centromere, finches, germline-restricted chromosome, GRC, хромосомная эволюция, флуоресцентная гибридизация in situ, центромера, вьюрки

## Abstract

An additional germline-restricted chromosome (GRC) has been found in the germline cells of all studied passerine bird species. It is eliminated from somatic cells during early embryogenesis and from spermatocytes after the first or second division of male meiosis. The GRC is transmitted across generations predominantly via the maternal line. It contains amplified and rearranged copies of genomic regions from the standard chromosome set. Some of these genes are expressed in the gonads of both males and females. However, the function and evolutionary dynamics of the GRC remain unknown. We conducted a comparative cytogenetic analysis of the GRC in five closely related finch species – the Eurasian bullfinch Pyrrhula pyrrhula, the common greenfinch Chloris chloris, the European goldfinch Carduelis carduelis, the common redpoll Acanthis flammea, and the pine grosbeak Pinicola enucleator – using fluorescent in situ hybridization (FISH) with a whole-chromosome DNA probe derived from the bullfinch GRC on spread spermatocytes of these species and immunolocalization of synaptonemal complex (SC) and centromere proteins. We described for the first time the SC karyotype of the pine grosbeak (2n = 82 + GRC). The standard chromosome set consists of nine submetacentric bivalents (seven macro- and two microbivalents) and 32 acrocentric microbivalents. All acrocentric microbivalents contain centromeres composed of multiple centromeric domains (metapolycentromeres). The grosbeak GRC is a large acrocentric macrounivalent. Cross-species in situ hybridization of the bullfinch GRC DNA probe showed only weak signals on the GRC of the grosbeak and redpoll, whereas no signal was detected on the greenfinch and goldfinch GRCs. These data are consistent with published results for two other representatives of this family and indicate rapid divergence and high species specificity of GRC sequences within the family Fringillidae. We also detected interspecies differences in the localization of sequences homologous to the bullfinch GRC on the bivalents of the standard set of these species. Thus, our data indicate rapid evolution of the GRC’s genetic composition and reveal species-specific dynamics of increase and decrease in the copy number of detected sequences in the standard chromosome set during the evolution of songbird species.

## Abstract (ru)

Добавочная хромосома, ограниченная клетками зародышевой линии (germline-restricted chromosome, GRC), обнаружена у всех исследованных воробьинообразных птиц. Она элиминируется из клеток соматической линии в раннем эмбриогенезе и из сперматоцитов после первого или второго деления мейоза. GRC передается в ряду поколений преимущественно по материнской линии, содержит амплифицированные и перестроенные копии последовательностей хромосом основного набора. Некоторые из них экспрессируются в гонадах самцов и самок. Однако функция и эволюционная динамика GRC остаются неизвестными. Мы провели сравнительный цитогенетический анализ GRC пяти видов птиц семейства Вьюрковые – обыкновенного снегиря Pyrrhula pyrrhula, обыкновенной зеленушки Chloris chloris, обыкновенного щегла Carduelis carduelis, обыкновенной чечётки Acanthis flammea и обыкновенного щура Pinicola enucleator – с использованием флуоресцентной гибридизации in situ ДНК-зонда к целой GRC обыкновенного снегиря с материалом ядер распластанных сперматоцитов данных видов и иммунолокализации белков синаптонемного комплекса и центромеры. Мы впервые описали кариотип синаптонемных комплексов обыкновенного щура (2n = 82 + GRC). Биваленты основного набора состоят из девяти субметацентрических (семь макро- и два микробивалента) и 32 акроцентрических микробивалентов. Все акроцентрические микробиваленты основного набора содержат центромеры, состоящие из нескольких центромерных доменов (метаполицентромеры). GRC щура представляет собой крупный акроцентрический макроунивалент. Перекрестная гибридизация in situ ДНК-зонда к GRC снегиря показала только слабые сигналы на GRC щура и чечётки, тогда как на GRC зеленушки и щегла сигналы отсутствовали. Эти данные согласуются с опубликованными результатами, полученными для двух других представителей этого семейства, и свидетельствуют о высокой видовой специфичности последовательностей GRC в пределах семейства Вьюрковые. Мы также обнаружили межвидовые различия в локализации последовательностей, сходных с последовательностями GRC снегиря, на бивалентах основного набора исследуемых видов. Таким образом, наши данные указывают на быструю эволюцию генетического состава GRC и видоспецифичную динамику увеличения и сокращения копийности выявленных последовательностей на хромосомах основного набора в ходе эволюции певчих птиц.

## Introduction

The Germline-Restricted Chromosome (GRC) has been identified in all studied passerine birds, indicating a monophyletic origin (Torgasheva et al., 2019). The GRC in all examined species contains amplified and rearranged copies of sequences from the standard (A) chromosomes. Some of these sequences are expressed in the gonads of both males and females (Biederman et al., 2018; Kinsella et al., 2019). The GRC is eliminated from somatic cell lineages during early embryogenesis and from spermatocytes after the first or second meiotic division (Pigozzi, Solari, 2005). The GRC is transmitted predominantly through the maternal line (Pei et al., 2022).

The elimination of the GRC from somatic cells allows genes located on it to escape the selective pressure characteristic of genes on the standard chromosomes. This is expected to lead to rapid changes in the GRC’s genetic composition. Indeed, the size and genetic content of the GRC vary widely among species. In some species, the GRC is one of the largest macrochromosomes (macro-GRC), while in others, it is one of the smallest microchromosomes (micro-GRC) (Borodin et al., 2022).

Results of cross-species fluorescent in situ hybridization (FISH) using whole-chromosome DNA probes derived from the GRC of the zebra finch (Taeniopygia guttata), the sand martin (Riparia riparia), the Eurasian siskin (Spinus spinus), and the great tit (Parus major) demonstrated an astonishingly low level of similarity between the GRCs of different species (the divergence time between the most distant species being ~25–30 million years) (Torgasheva et al., 2019, 2021). This indicates extremely rapid evolution of this chromosome’s genetic content. This conclusion is supported by comparative sequencing data of the micro-GRC in closely related species, such as the thrush nightingale (Luscinia luscinia) and the common nightingale (L. megarhynchos), which revealed substantial differences in GRC composition despite their relatively recent divergence (approximately 1.8 million years ago) (Schlebusch et al., 2023).

Many genes on the GRC are present in a fragmented, likely non-functional state, with the exception of a small number of highly conserved and presumably essential genes. This rapid change in genetic composition and the abundance of duplications, deletions, and pseudogenes stand in sharp contrast to the generally conserved avian karyotype, making the GRC the most rapidly evolving chromosome in the passerine bird genome (Borodin et al., 2022).

The rapid changes in the size and genetic composition of the GRC and the consequent diversity of its gene content – including genes involved in reproductive system development – have prompted the formulation of several hypotheses. One posits that the GRC resolves germline-soma conflict by isolating genes with mutually antagonistic effects. Another hypothesis views the GRC as a highly efficient genomic parasite. A third considers this chromosome a potential driver of speciation, as rapid divergence of its genetic content in isolated populations is expected to lead to genetic incompatibilities. In our view, these hypotheses are not mutually exclusive. It is highly probable that the GRC fulfills all of these roles simultaneously (Borodin et al., 2022; Borodin, 2023; Vontzou et al., 2023).

The scale and patterns of GRC variability at the cytogenetic level within narrow taxonomic groups, such as a family, remain insufficiently studied. Cross-species FISH experiments comparing GRC genetic content within a single family have so far encompassed only two or three species (Torgasheva et al., 2019). A more systematic comparative analysis of the GRC across multiple members of a single clade has not been previously conducted

**Fig. 1.**
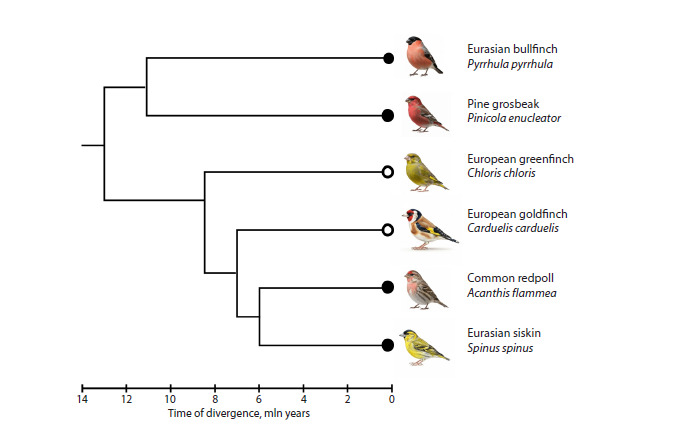
A cladogram of bird species from the family Fringillidae used for the comparative analysis of the GRC. A black circle denotes a macro-GRC, a white circle denotes a micro-GRC. The cladogram was constructed using the Timetree.org resource (Kumar et al., 2017) (last accessed December 3, 2025).

The aim of the present work was a comparative cytogenetic analysis of the GRC in five species of the family Fringillidae and an assessment of the pattern of in situ hybridization of a DNA probe derived from the whole macro-GRC of the Eurasian bullfinch (Pyrrhula pyrrhula) with nuclear spreads of spermatocytes of several species with varying degrees of phylogenetic relatedness and different GRC morphology. For this purpose, we selected four species representing different genera within this family (Fig. 1): the European greenfinch (Chloris chloris) (micro-GRC), the European goldfinch (Carduelis carduelis) (micro-GRC), the common redpoll (Acanthis flammea) (macro-GRC), and the pine grosbeak (Pinicola enucleator), the karyotype and GRC morphology of which had not been described until now.

The selected species represent different evolutionary lineages within the family: the divergence time between the bullfinch and the pine grosbeak is approximately 11 million years, and between the bullfinch and the greenfinch, goldfinch, and redpoll, it is approximately 13 million years (Price et al., 2014; Hooper, Price, 2017). Integrating the results of this experiment with data previously obtained from the analysis of two other species in this family – the Eurasian siskin (Spinus spinus) (macro-GRC) and the European goldfinch (Carduelis carduelis) (micro-GRC) (Torgasheva et al., 2019), which diverged approximately 8.5 million years ago (Price et al., 2014; Hooper, Price, 2017), – will allow for a more detailed assessment of GRC variability within a single family.

## Materials and methods

Biological material was obtained from birds delivered with fatal injuries to the Wildlife Rehabilitation Center (Novosibirsk) between April and May 2022–2023. This study examined one male of each species: the Eurasian bullfinch (Pyrrhula pyrrhula), pine grosbeak (Pinicola enucleator), European greenfinch (Chloris chloris), European goldfinch (Carduelis carduelis), and common redpoll (Acanthis flammea). Euthanasia was performed by isoflurane overdose (Laboratories Karizoo, S.A., Barcelona, Spain). Handling of birds and euthanasia were conducted in accordance with national regulations for the care and use of laboratory animals. The experimental protocol was reviewed and approved by the Bioethics Commission of the Institute of Cytology and Genetics, Siberian Branch of the Russian Academy of Sciences (Protocols No. 114 dated December 17, 2021, and No. 199 dated November 21, 2024).

Synaptonemal complex (SC) spreads were prepared according to the method of A.H. Peters et al. (1997). Immunocytochemical detection of SC proteins and centromeres was performed following the protocol of L.K. Anderson et al. (1999) using the following primary antibodies: rabbit polyclonal antibodies to SYCP3 (dilution 1:500; Abcam, UK; cat. No. ab15093), anti-centromere antibodies from the serum of patients with CREST syndrome (dilution 1:100; Antibodies Inc., USA; cat. No. 15-234). The following secondary antibodies were used: goat anti-rabbit IgG conjugated with Cy3 (dilution 1:500; Jackson ImmunoResearch, USA; cat. No. 111-165-144), donkey anti-human IgG conjugated with AMCA (dilution 1:100; Jackson ImmunoResearch, USA; cat. No. 709-155-149).

Slides were incubated overnight at +4 °C with primary antibodies and for one hour at +37 °C with secondary antibodies in a humid chamber. To prevent photobleaching, Vectashield mounting medium (Vector Laboratories, USA; cat. No. H-1000-10) was applied to the slides.

The DNA probe for the whole bullfinch GRC was obtained by microdissection of five micronuclei copies from meiotic chromosome spreads, as described by A.A. Torgasheva et al. (2019). Slides were preliminarily stained with a 0.1 % Giemsa solution (Sigma, USA) for 3–5 minutes at room temperature. DNA from the microdissected micronuclei was amplified and labeled with biotin-11-dUTP (Sigma, USA) using the Genome- Plex Whole Genome Amplification Kit (Sigma-Aldrich, USA; cat. No. WGA1).

FISH using the bullfinch GRC DNA probe was performed according to a standard protocol (Liehr et al., 2017) with some modifications. The hybridization mixture (32 μl) contained hybridization buffer (50 % formamide, 2× SSC), 0.2 % Tween 20, and 40 ng of labeled probe. DNA on slides pretreated with RNase A was denatured in 70 % formamide with 2× SSC at +72 °C for 3 minutes. The probe was denatured at +95 °C for 5 minutes. Hybridization was carried out overnight at +39 °C in a humid chamber. The biotin-labeled probe was detected using avidin-FITC (dilution 1:400) and anti-avidin-FITC (dilution 1:200) (Vector Laboratories, USA). Slides were mounted in Vectashield medium with DAPI (Vector Laboratories, USA, cat. No. H-1200-10).

Images of SCs after immunolocalization and FISH were captured using a CCD camera mounted on an Axioplan 2 microscope (Carl Zeiss, Germany) with filters No. 49 (DAPI), 10 (FITC), and 15 (TRITC) (ZEISS, Germany) and ISIS4 software (METASystems GmbH, Germany). Brightness and contrast of the images were adjusted using Corel PaintShop Photo Pro X6 (Alludo, Canada).

For the construction of the SC karyotype idiogram for the pine grosbeak, 28 cells were measured. The length of SCs and the position of centromeres were determined in micrometers using the MicroMeasure 3.3 program (Reeves, 2001). All raw data are provided in the Supplementary Material^1^. In each cell, SCs were ranked by relative length and centromeric index, and average values across all cells were calculated.

Supplementary Materials are available in the online version of the paper: https://vavilov.elpub.ru/jour/manager/files/Suppl_Mal_Engl_30_3.xlsx


Descriptive statistics were obtained using Statistica 6.0 (StatSoft Inc., Tulsa, OK, USA, 2001). The text presents values as mean ± standard deviation.

## Results

**Fig. 2.**
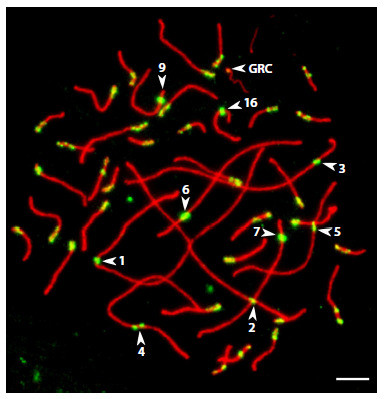
Photograph of an SC spread from a pine grosbeak pachytene spermatocyte after immunolocalization of the SYCP3 protein (red) and centromeric proteins (green). Arrowheads indicate submetacentric bivalents 1–7, 9, 16, and the GRC. Scale bar = 5 μm.

**Fig. 3.**
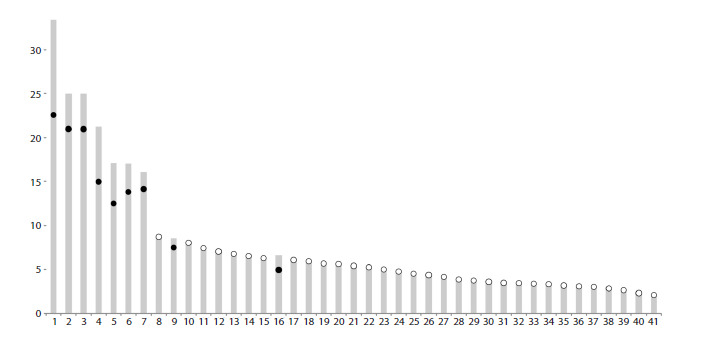
SC idiogram of the pine grosbeak karyotype, excluding the GRC. The Y axis shows the average SC length in μm. The X axis shows bivalents ordered by decreasing size. Black circles indicate the location of monocentromeres, white circles indicate metapolycentromeres.

Neither the somatic nor the synaptonemal complex (SC) karyotype of the pine grosbeak (Pinicola enucleator L.) had been previously described. We found that the SC karyotype of this species comprises 41 pairs of bivalents from the standard set and a GRC (2n = 82 + GRC; fundamental number (FN) = 50) (Fig. 2). The total SC length was 334 ± 47 μm. Macrobivalents 1–7, as well as microbivalents 9 and 16, are submetacentric; microbivalents 8 and all other microbivalents are acrocentric (Fig. 3). All acrocentric microbivalents of the standard set contain centromeres composed of multiple centromeric domains. Such centromeres are conventionally referred to as metapolycentromeres (Grishko, Borodin, 2024). The GRC of the pine grosbeak is a large acrocentric macrochromosome. At the pachytene stage, it forms an acrocentric univalent that is recognized by antibodies against the SYCP3 protein, which forms the lateral element of the SC (Fig. 2).

To assess the similarity between the GRC sequences of the bullfinch and the GRCs of other finch species, cross-species FISH was performed using a DNA probe for the bullfinch GRC obtained previously (Grishko et al., 2025). The probe hybridized weakly to the macro-GRC of the pine grosbeak and common redpoll, and did not hybridize to the micro-GRC of the European greenfinch and European goldfinch (see the Table).

**Table 1.**
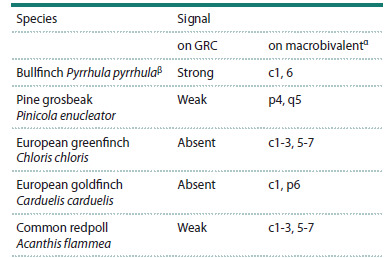
Detection of hybridization signal after FISH with the bullfinch GRC DNA probe on SC spreads of finch species α с – centromere, p – short arm of the bivalent, q – long arm of the bivalent. β (Grishko et al., 2025).

**Fig. 4.**
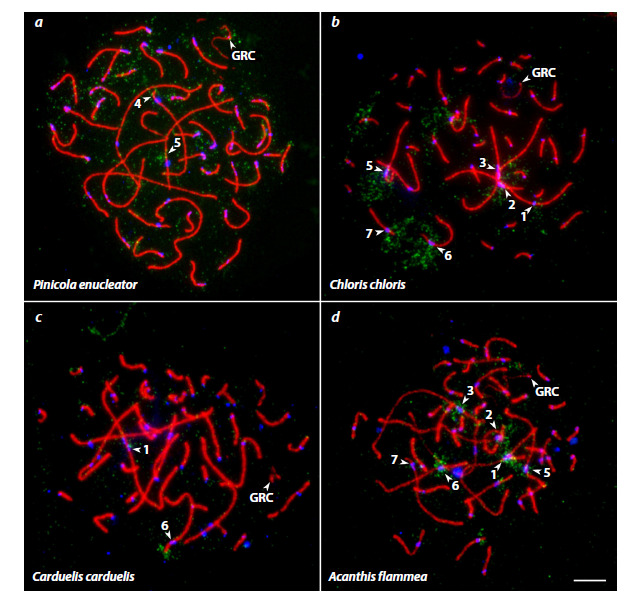
Photographs of SC spreads from pachytene spermatocytes of the pine grosbeak (a), European greenfinch (b), European goldfinch (c), and common redpoll (d) after FISH with the bullfinch GRC DNA probe (green), immunolocalization of the SYCP3 protein (red), and centromeric proteins (blue). Arrowheads indicate the GRC and standard bivalents labeled by the bullfinch GRC DNA probe. Scale bar = 5 μm.

In all four species, the probe labeled several regions on the standard bivalents. In SC spreads from the pine grosbeak, hybridization signals were observed on the short arm of macrobivalent 4 and in the long arm region of macrobivalent 5 (Fig. 4a). The probe also produced weak signals on many other bivalents. In SC spreads from the European greenfinch and common redpoll, the probe hybridized to the pericentromeric regions of several microbivalents and all macrobivalents, with the exception of a single metacentric macrobivalent (likely ZZ) (Fig. 4b, d). In SC spreads from the European goldfinch, the hybridization signal was detected in the pericentromeric region of macrobivalent 1 and on the short arm of macrobivalent 6 (Fig. 4c).

## Discussion

In the present study, we have described, for the first time, the synaptonemal complex (SC) karyotype of the pine grosbeak, demonstrating that it, like the karyotypes of half of the studied finch species (Borodin et al., 2022; Malinovskaya et al., 2022), contains a macro-GRC. We established that many bivalents in this species possess metapolycentromeres. The presence of metapolycentromeres was previously demonstrated in the Eurasian bullfinch and the common linnet (Linaria cannabina) from the same family, Fringillidae (Grishko et al., 2023).

The primary result of this investigation is the detection of high interspecific variability in the genetic composition of the GRC among representatives of the family Fringillidae. Despite the relatively close phylogenetic relatedness among these finches (with divergence time between the bullfinch and the other studied species ranging from approximately 11 to 13 million years (Price et al., 2014; Hooper, Price, 2017)), the DNA probe for the bullfinch macro-GRC yielded a weak hybridization signal on the macro-GRC of the pine grosbeak and common redpoll and produced no detectable signal on the micro-GRC of the European greenfinch and European goldfinch.

The absence of a detectable signal on the micro-GRC of the greenfinch and goldfinch, in contrast to the weak signal on the macro-GRC of the pine grosbeak and redpoll, suggests that GRC size may be one of the factors influencing the degree of detectable sequence similarity, at least within a single family. This pattern aligns with data from the literature: for instance, with a shorter divergence time (~9 million years), a DNA probe for the micro-GRC of the Eurasian siskin produced a weak hybridization signal on the macro-GRC of the European goldfinch (Torgasheva et al., 2019). It is likely that over ~9–13 million years of divergence, micro-GRCs lost most of their shared sequences, resulting in the absence of a hybridization signal, whereas macro-GRCs retained a sufficient number of homologous sequences to produce a weak signal.

In our cross-species FISH experiments, we identified differences in the hybridization patterns of the bullfinch GRC DNA probe with the standard bivalents among different finch species. It was previously shown that this probe hybridized to the pericentromeric regions of specific macrobivalents and a number of microbivalents in the bullfinch (Grishko et al., 2025). In our cross-species FISH experiments, this probe demonstrated a different hybridization pattern. The observed interspecific differences in hybridization patterns indicate rapid evolution of the repetitive sequences located on the standard chromosomes, occurring against the backdrop of the overall conservation of avian genomes.

The hybridization of the bullfinch GRC DNA probe to the pericentromeric regions of all macrobivalents, except macrobivalent 4, in the greenfinch and redpoll may indicate the conservation of these sequences in these two species. This pattern contrasts with that observed in the pine grosbeak and goldfinch, where the probe hybridized specifically only to distinct regions of certain macrobivalents. This points to a more limited and species-specific distribution of the detectable sequences across the standard bivalents in these species. The weak diffuse signals detected on most bivalents in the pine grosbeak may represent “ghost” sequences that were once more widely distributed but have subsequently degraded or been replaced in most genomic regions.

Thus, the GRC is a rapidly evolving genomic element in passerine birds, showing a low degree of similarity even among species within the single family Fringillidae. The differences in the hybridization pattern of the bullfinch GRC DNA probe on spermatocyte nuclear spreads from different finch species indicate a species-specific dynamic of amplification and reduction in the copy number of the detectable sequences on the standard bivalents over the course of evolution.

## Conclusion

Our study has demonstrated that the genetic composition of the germline-restricted chromosome (GRC) in individual representatives of the family Fringillidae is highly speciesspecific, indicating rapid evolution of the GRC. The presence of shared sequences between the bullfinch GRC and the standard bivalents of different finch species, coupled with the species-specific patterns of their localization, aligns with similar results from cross-species FISH experiments utilizing DNA probes derived from the GRC of other bird species.

## Conflict of interest

The authors declare no conflict of interest.
